# Leisure Time Physical Activity, Sedentary Time in Pregnancy, and Infant Weight at Approximately 12 Months

**DOI:** 10.1089/whr.2020.0068

**Published:** 2020-05-12

**Authors:** Sylvia E. Badon, Alyson J. Littman, Kwun Chuen Gary Chan, Michelle A. Williams, Helene Kirkegaard, Ellen A. Nohr, Daniel A. Enquobahrie

**Affiliations:** ^1^ Department of Epidemiology, University of Washington, Seattle, Washington, USA.; ^2^ Division of Research, Kaiser Permanente Northern California, Oakland, California, USA.; ^3^ Seattle Epidemiologic Research and Information Center, Department of Veterans Affairs Puget Sound Health Care System, Seattle, Washington, USA.; ^4^ Department of Biostatistics, University of Washington, Seattle Washington, USA.; ^5^ Department of Epidemiology, Harvard T.H. Chan School of Public Health, Boston, Massachusetts, USA.; ^6^ Research Unit of Obstetrics and Gynecology, Department of Clinical Research, University of Southern Denmark, Odense, Denmark.

**Keywords:** infant weight, physical activity, pregnancy, sedentary time

## Abstract

***Background:*** Maternal leisure time physical activity (LTPA) and sedentary time during pregnancy may influence programming of infant growth in a sex-specific manner.

***Materials and Methods:*** Participants (*N* = 35,212) from the Danish National Birth Cohort reported moderate/vigorous LTPA (hours/week) in early (conception to mean 16 weeks of gestation) and late pregnancy (mean 31 weeks of gestation to delivery) during interviews at 16 weeks of gestation and 6 months postpartum. Participants reported screen time at work and time spent watching television/videos (hours/day) in early pregnancy. Infant weight at 12 months of age was reported by mothers. Weight-for-length was categorized using sex-specific international standards.

***Results:*** Participants reported on average 1 hour per week of early pregnancy moderate/vigorous LTPA, 0.5 hour per week of late pregnancy LTPA, and 3 hours per day of early pregnancy sedentary time. Early pregnancy LTPA category was not associated with infant weight (*p* for trend = 0.62). There were suggested associations of early pregnancy sedentary time above the first quartile with greater odds of infant underweight (odds ratio = 1.15–1.27; *p* for trend = 0.27). Associations were similar in male and female infants.

***Conclusions:*** There is no clear relationship between early or late pregnancy LTPA and infant weight at 12 months in our study. Maternal early pregnancy sedentary time may be associated with infant underweight at 12 months.

## Introduction

Growth in infancy is associated with future risk of cardiovascular and metabolic diseases, including coronary heart disease^1^ and glucose intolerance^2^ over the life course. Factors that determine growth in infancy, particularly those related to maternal perinatal characteristics, have not been fully described. Maternal factors during the perinatal period such as maternal leisure time physical activity (LTPA) and sedentary behavior may have long-term effects on offspring postnatal growth and health directly through epigenetic mechanisms^3–6^ or indirectly through reduction of pregnancy complications^7–9^ and fetal overgrowth.^10–12^

Previous studies of maternal LTPA during pregnancy and offspring growth have focused on growth during childhood (5–8 years old).^13,14^ Results from these studies suggest that maternal LTPA throughout pregnancy is associated with a decrease in offspring weight and weight-for-length at ages 5–8 years. Previous studies have not investigated associations of maternal LTPA or sedentary behavior with offspring weight at younger ages. Programming effects that are identified in early postnatal life may be more easily and effectively intervened upon, compared with later life exposures, to influence adolescent and adult health.

Previous research also suggests sex-specific placental responses to the intrauterine environment^15^ and sex-specific epigenetic changes in maternal blood in response to physical activity during pregnancy,^6^ which may affect fetal programming of infant growth in a sex-specific manner. Reports of sex-specific differences in patterns and determinants of fetal and infant growth^16^ and in associations of maternal LTPA with infant birth size^10^ have also been reported. However, previous studies have not examined potential sex-specific differences in postnatal growth in relation to LTPA and sedentary behavior during pregnancy. The objective of this study was to investigate associations of maternal LTPA and sedentary time during early and late pregnancy, both critical periods for fetal programming of growth,^17^ with infant weight at ∼12 months, and whether these associations differ by infant sex.

## Materials and Methods

### Study setting and study population

This cohort study was conducted using data from the Danish National Birth Cohort (DNBC),^18^ a prospective pregnancy cohort study designed to examine early life risk factors of pregnancy complications and offspring outcomes. From 1996 to 2002, pregnant women in Denmark were recruited for this study by general practitioners at their first prenatal visit. All pregnant women in Denmark who were able to speak Danish well enough to participate in telephone interviews and planned to carry the pregnancy to term were eligible. A total of 91,386 women with 100,418 pregnancies (∼35% of the eligible population) participated in the study. All participants gave written informed consent. The DNBC was approved by the Scientific Ethics Committee in Denmark and the Danish Data Protection Agency, which also approved the present study.

Participants in the DNBC agreed to complete four telephone interviews: two during pregnancy at an average of 16 (range: 11–25) and 31 (range: 27–37) weeks of gestation and two after delivery, when the child was ∼6 and 18 months old. Information collected during interviews included sociodemographic characteristics, reproductive and medical history, LTPA and sedentary time during pregnancy, breastfeeding, and infant weight.

Live, singleton births (*N* = 92,664) were eligible for the current analyses. Participants who had offspring with conditions affecting growth such as chromosomal abnormalities (*N* = 53) and congenital malformations (*N* = 2,433) were excluded. For women with more than one birth in the cohort (*N* = 6,033), the first birth in the study was selected. Women who did not participate in interview 4 (*N* = 18,674), during which data on infant weight at ∼12 months were collected, were also excluded. Of the 59,950 women who participated in interview 4, those with missing data on infant weight (*N* = 13,621), maternal perinatal LTPA (*N* = 1,648), sedentary behavior (*N* = 25), smoking (*N* = 7,425), prepregnancy body mass index (BMI, *N* = 561), or other included covariates (*N* = 450) were also excluded. In addition, participants with LTPA >35 hours per week (*N* = 3) or a 12-month growth measurement before 9 or after 15 months of age (*N* = 1,005) were excluded. A total of 35,212 women were included in analyses. Selected characteristics of participants included in the current analyses and participants excluded from the current analyses were evaluated and found to be similar (Supplementary Table S1).

### Leisure time physical activity

During interviews 1 and 3, participants were asked to report any moderate/vigorous LTPA (aerobics/gymnastics, dancing, cycling, fast walking, jogging, ball games, swimming, fitness classes, badminton, tennis, horseback riding, or other activities) during early pregnancy [from conception to mean 16 weeks of gestation (range: 11–25)] and late pregnancy [from mean 31 weeks of gestation (range: 27–37) to delivery]. For each activity reported, the average frequency per week and average duration of each episode of LTPA were recorded. Average LTPA duration per week during early pregnancy, and separately for late pregnancy, was calculated by summing duration per week of reported physical activities.

### Sedentary time

During interview 1, participants were asked to report the average number of hours spent watching television or videos per day. Response options for time spent watching television or videos ranged from <1 hour to 5 or more hours per day in 0.5-hour increments. Participants also reported average number of hours spent working with a computer screen during interview 1. Responses for time spent watching television or videos and responses for time spent working with a computer screen were summed to calculate total daily sedentary time.

### Infant weight

During interview 4, mothers reported weight to the nearest gram and length to the nearest 0.5 cm of their children around 12 months of age (range: 9–15 months), as measured by their general practitioner during routine childhood care. Weight-for-length at ∼12 months was categorized as underweight (<5th percentile), normal weight (5th–84th percentile), overweight (85th–94th percentile), or obese (≥95th percentile) using the World Health Organization sex-specific international standards for weight-for-length.^19^

### Covariates

Sociodemographic characteristics, reproductive and medical history, prepregnancy weight, and height were collected during interview 1. Breastfeeding information was collected during interview 4. Maternal prepregnancy BMI, calculated using self-reported height and weight, was categorized using standard cut points: underweight: <18.5 kg/m^2^, normal weight: 18.5–24.9 kg/m^2^, overweight: 25–29.9 kg/m^2^, obese: ≥30 kg/m^2^.^20^ Parental socio-occupational status was determined using reported employment information for mothers and their partners obtained at the early pregnancy interview and categorized into “high,” “middle,” or “low” as previously used in studies using data from the DNBC.^21^ Participants also reported whether their job was physically demanding. Duration of any breastfeeding was categorized as 0–13, 14–21, and ≥22 weeks.

### Statistical analyses

Means, standard deviations, medians, and interquartile ranges were used to describe continuous variables. Frequencies and percentages were used to describe categorical variables.

Linear regression was used to determine mean differences and corresponding 95% confidence intervals (CIs) in weight, adjusted for height, at ∼12 months for each additional hour of early or late pregnancy LTPA or early pregnancy sedentary time. LTPA and sedentary time were also treated as categorical exposures. Early and late pregnancy LTPA was categorized into four groups: all women who reported no LTPA (0 minutes/week) were one group, and LTPA among women who reported any LTPA was categorized into tertiles. Sedentary time was categorized into quartiles. Generalized logistic regression models (baseline-category logit models^22^) were used to calculate odds ratios (ORs) and 95% CIs for underweight, overweight, or obese compared with normal weight at ∼12 months. LTPA and sedentary time categories were also modeled as continuous variables to determine *p* values for linear trend. The model was adjusted for *a priori* selected confounders and precision variables: maternal age (years), prepregnancy BMI category (underweight/normal weight/overweight/obese), nulliparity (yes/no), smoking during pregnancy (yes/no), spouse/partner (yes/no), parental socio-occupational status (low/middle/high), employment category (working/on sick leave/on other leave/student/unemployed), infant age at reported 12-month measurement during interview 4 (months), infant length at reported 12-month measurement during interview 4 (cm), and infant sex. Regression coefficients for LTPA and sedentary time were estimated from one model. We also conducted sensitivity analyses with early and late pregnancy LTPA in one model, additional adjustment for a measure of general occupational activity (self-reported physically demanding job), additional adjustment for duration of any breastfeeding (0–13, 14–21, and ≥22 weeks), and using 1-hour categories of LTPA as the exposure.

Two-way multiplicative interaction terms and corresponding *p* values were used to assess interactions of early or late pregnancy LTPA and early pregnancy sedentary time with infant sex. All analyses were performed using SAS 9.4 (SAS Institute, Inc., Cary, NC).

## Results

Participants reported ∼1 hour per week of early pregnancy LTPA and 0.5 hour per week of late pregnancy LTPA, on average (Table 1). About two-thirds of participants were normal weight, and about half were of high socio-occupational status. Fifty percent of participants were nulliparous. Infants weighed 10.2 kg at ∼12 months of age, on average. Women with high early pregnancy LTPA were more likely to be normal weight, have high socio-occupational status, be nulliparous, not smoke during pregnancy, and have a longer duration of breastfeeding compared with women with no early pregnancy LTPA (Supplementary Table S2). Early pregnancy and late pregnancy LTPA were weakly correlated (Pearson *r* = 0.27). Women with high sedentary time (≥5 hours/day) were more likely to have middle socio-occupational status, be employed and working, and be nulliparous, compared with women with low sedentary time (Supplementary Table S3). Among women who were employed, women with high sedentary time were less likely to have an occupation that was physically demanding and more likely to have an occupation that involved mostly sitting than women with low sedentary time.

**Table 1.** Maternal and Infant Characteristics, Danish National Birth Cohort 1997–2003

Maternal characteristics	Overall	
	N	Median (IQR)
Age (years), mean (SD)	35,212	30.2 (4.2)
Early pregnancy moderate/vigorous leisure time physical activity (hours/week)	35,212	0.0 (0.0–1.0)
Late pregnancy moderate/vigorous leisure time physical activity (hours/week)	35,130	0.0 (0.0–0.0)
Early pregnancy total sedentary time (hours/day)	35,212	2.4 (1.5–4.6)
	*N*	%
Any early pregnancy moderate/vigorous leisure time physical activity	13,132	37
Any late pregnancy moderate/vigorous leisure time physical activity	8,730	25
Prepregnancy BMI category		
Underweight (<18.5 kg/m^2^)	1,422	4
Normal weight (18.5–24.9 kg/m^2^)	23,699	67
Overweight (25–29.9 kg/m^2^)	7,082	20
Obese (≥30 kg/m^2^)	3,009	9
Spouse/partner	34,768	99
Socio-occupational status		
Low	2,890	8
Middle	13,774	39
High	18,548	53
Nulliparous	17,545	50
Smoked during pregnancy	8,597	24
Gestational diabetes (*n* = 35,101)	309	1
Preeclampsia	804	2
Total breastfeeding duration (weeks)		
0–13	7,518	21
14–21	3,952	11
≥22	23,742	67
Infant characteristics	*N*	Mean (SD)
Birthweight (g)	35,212	3,596 (548)
Gestational age at delivery (weeks)	35,212	39.6 (1.7)
Reported weight at ∼12 months of age^a^ (kg)	35,212	10.2 (1.2)
Reported age at interview 4^a^ (months)	35,212	12.4 (0.6)
	*N*	%
Male sex	17,729	50
Weight-for-length category at ∼12 months^a^		
Underweight (<5th percentile)	818	2
Normal weight (5th–84th percentile)	24,252	69
Overweight (85th–94th percentile)	5,564	16
Obese (≥95th percentile)	4,509	13

^a^ Range = 9–15 months.

BMI, body mass index; IQR, interquartile range; SD, standard deviation.

### Early and late pregnancy LTPA

Overall, early pregnancy LTPA was not associated with weight at ∼12 months [mean difference = 0.00 kg; 95% CI: −0.01 to 0.00] (Table 2). There was a trend for associations between increasing early pregnancy LTPA tertile and lower odds of overweight (*p* for trend = 0.08). Moderate levels (tertile 2) of early pregnancy LTPA were associated with 12% lower odds of obesity (95% CI: 0.78 to 0.98). High levels of early pregnancy LTPA (tertile 3) were not associated with obesity at 12 months (OR = 0.99, 95% CI: 0.89 to 1.09). There was a similar trend for associations between increasing late pregnancy LTPA tertile and lower odds of overweight (*p* for trend = 0.11), but no associations of late pregnancy LTPA with obesity (Supplementary Table S4). Associations did not differ by infant sex (all *p* for interaction >0.05). Results were similar after adjusting early pregnancy LTPA models for later pregnancy LTPA (Supplementary Table S5).

**Table 2.** Associations of Early Pregnancy Leisure Time Physical Activity (Hours/Week) with Infant Weight at ∼12 Months of Age

	Weight (kg) adjusted for length (cm)		Underweight (<5th percentile)		Normal weight (5–84th percentile)		Overweight (85–94th percentile)		Obese (≥95th percentile)	
	N	Mean difference (95% CI)	N	OR (95% CI)^a^	N	OR (95% CI)^a^	N	OR (95% CI)^a^	N	OR (95% CI)^a^
Continuous (hours/week)	35,212	0.00 (−0.01 to 0.00)	818	0.99 (0.95 to 1.04)	24,252	Ref.	5,564	0.98 (0.97 to 1.00)	4,509	0.99 (0.97 to 1.01)
No leisure time physical activity	22,080	Ref.	509	Ref.	15,111	Ref.	3,521	Ref.	2,886	Ref.
Tertile 1 (0.01–1.00)	5,003	0.00 (−0.03 to 0.03)	125	1.08 (0.89 to 1.32)	3,434	Ref.	821	1.03 (0.95 to 1.12)	620	0.96 (0.87 to 1.06)
Tertile 2 (1.05–2.23)	3,752	−0.04 (−0.07 to 0.00)	83	0.92 (0.73 to 1.17)	2,659	Ref.	562	0.91 (0.83 to 1.01)	441	0.88 (0.79 to 0.98)
Tertile 3 (2.25–30)	4,377	0.01 (−0.03 to 0.04)	101	0.98 (0.79 to 1.22)	3,048	Ref.	660	0.94 (0.86 to 1.03)	562	0.99 (0.89 to 1.09)
*p* for trend		0.62		0.73				0.08		0.21

*p* for interaction with infant sex: continuous LTPA: weight *p* = 0.72; weight categories *p* = 0.55; LTPA tertiles: weight *p* = 0.91; weight categories *p* = 0.98.

Range = 9–15 months.

Model adjusted for maternal age (years), prepregnancy BMI category (underweight/normal weight/overweight/obese), nulliparity (yes/no), smoking during pregnancy (yes/no), spouse/partner (yes/no), socio-occupational status (high/middle/low), employment (working/on sick leave/on other leave/student/unemployed), total sedentary time (hours/day), infant age at interview 4 measurement (months), infant length at interview 4 measurement (cm), and infant sex.

^a^ Generalized logistic regression model with normal weight as the reference group.

CI, confidence interval; LTPA, leisure time physical activity; OR, odds ratio.

### Early pregnancy sedentary time

Maternal sedentary time during early pregnancy was not associated with infant weight at ∼12 months of age [mean difference = 0.00 kg; 95% CI: −0.01 to 0.00] (Table 3). There were suggested associations of early pregnancy sedentary time levels above the first quartile with greater odds of underweight at ∼12 months of age (OR = 1.27; 95% CI: 1.04 to 1.55 for quartile 2, OR = 1.15; 95% CI: 0.93 to 1.42 for quartile 3, and OR = 1.19; 95% CI: 0.96 to 1.48 for quartile 4).

**Table 3.** Associations of Early Pregnancy Sedentary Time with Infant Weight at ∼12 Months

Early pregnancy sedentary time (hours/day)	Weight (kg) adjusted for length (cm)		Underweight (<5th percentile)		Normal weight (5–84th percentile)		Overweight (85–94th percentile)		Obese (≥95th percentile)	
	N	Mean difference (95% CI)	N	OR (95% CI)^a^	N	OR (95% CI)^a^	N	OR (95% CI)^a^	N	OR (95% CI)^a^
Continuous	35,212	0.00 (−0.01 to 0.00)	818	1.01 (0.98 to 1.05)	24,252	Ref.	5,564	1.00 (0.98 to 1.01)	4,509	0.99 (0.98 to 1.01)
Quartile 1 (0–1.4)	7,978	Ref.	163	Ref.	5,520	Ref.	1,278	Ref.	1,001	Ref.
Quartile 2 (1.5–2.3)	9,636	−0.01 (−0.04 to 0.02)	245	1.27 (1.04 to 1.55)	6,614	Ref.	1,509	0.97 (0.89 to 1.05)	1,249	1.01 (0.93 to 1.11)
Quartile 3 (2.4–4.5)	8,719	−0.01 (−0.04 to 0.03)	200	1.15 (0.93 to 1.42)	5,998	Ref.	1,342	0.94 (0.86 to 1.03)	1,160	1.04 (0.95 to 1.15)
Quartile 4 (4.6–10.7)	8,879	−0.01 (−0.05 to 0.02)	210	1.19 (0.96 to 1.48)	6,120	Ref.	1,435	0.99 (0.90 to 1.08)	1,099	0.99 (0.90 to 1.09)
*p* for trend		0.47		0.27				0.65		0.98

*p* for interaction with infant sex: continuous sedentary behavior: weight *p* = 0.59; weight categories *p* = 0.92; sedentary behavior quartiles: weight *p* = 0.03; weight categories *p* = 0.06.

Range = 9–15 months.

Model is adjusted for maternal age (years), prepregnancy BMI category (underweight/normal weight/overweight/obese), nulliparity (yes/no), smoking during pregnancy (yes/no), spouse/partner (yes/no), socio-occupational status (low/middle/high), employment (working/on sick leave/on other leave/student/unemployed), infant sex, infant age at ∼12-month measurement (months), and maternal early pregnancy LTPA (hours/week).

^a^ Generalized logistic regression model with normal weight as the reference group.

### Differences by infant sex

Interaction terms between total sedentary time and infant sex suggested different associations of early pregnancy sedentary time quartile with infant weight and weight categories by infant sex (*p* for interaction = 0.03 for continuous weight, 0.06 for weight categories). In stratified analyses by infant sex, there were no clear differences in associations of early pregnancy sedentary time with infant weight or weight categories (Table 4).

**Table 4.** Associations of Early Pregnancy Sedentary Time with Infant Weight at ∼12 Months by Sex

	Males		Females	
	N	Mean difference (95% CI)	N	Mean difference (95% CI)
Weight (kg) adjusted for length (cm)				
Quartile 1 (0–1.4)	4,035	Ref.	3,943	Ref.
Quartile 2 (1.5–2.3)	4,853	−0.01 (−0.05 to 0.04)	4,783	−0.01 (−0.05 to 0.03)
Quartile 3 (2.4–4.5)	4,382	−0.03 (−0.08 to 0.01)	4,337	0.02 (−0.02 to 0.06)
Quartile 4 (4.6–10.7)	4,459	0.01 (−0.04 to 0.06)	4,420	−0.04 (−0.08 to 0.01)
*p* for trend		0.99		0.26
	*N*	OR (95% CI)^a^	*N*	OR (95% CI)^a^
Underweight (<5th percentile)				
Quartile 1 (0–1.4)	94	Ref.	69	Ref.
Quartile 2 (1.5–2.3)	145	1.31 (1.01 to 1.71)	100	1.22 (0.89 to 1.66)
Quartile 3 (2.4–4.5)	130	1.27 (0.97 to 1.67)	70	0.98 (0.69 to 1.37)
Quartile 4 (4.6–10.7)	123	1.16 (0.87 to 1.54)	87	1.26 (0.90 to 1.77)
*p* for trend		0.44		0.41
Overweight (85–94th percentile)				
Quartile 1 (0–1.4)	677	Ref.	601	Ref.
Quartile 2 (1.5–2.3)	778	0.95 (0.85 to 1.07)	731	0.98 (0.87 to 1.10)
Quartile 3 (2.4–4.5)	717	0.97 (0.86 to 1.09)	625	0.91 (0.81 to 1.04)
Quartile 4 (4.6–10.7)	727	0.98 (0.87 to 1.11)	708	0.99 (0.87 to 1.13)
*p* for trend		0.82		0.67
Obese (≥94th percentile)				
Quartile 1 (0–1.4)	561	Ref.	440	Ref.
Quartile 2 (1.5–2.3)	708	1.05 (0.92 to 1.18)	541	0.97 (0.85 to 1.11)
Quartile 3 (2.4–4.5)	594	0.99 (0.87 to 1.12)	566	1.11 (0.97 to 1.27)
Quartile 4 (4.6–10.7)	633	1.08 (0.95 to 1.23)	466	0.89 (0.77 to 1.03)
*p* for trend		0.45		0.43

Range = 9–15 months.

Model is adjusted for maternal age (years), prepregnancy BMI category (underweight/normal weight/overweight/obese), nulliparity (yes/no), smoking during pregnancy (yes/no), spouse/partner (yes/no), socio-occupational status (low/middle/high), employment (working/on sick leave/on other leave/student/unemployed), infant sex, infant age at ∼12-month measurement (months), and maternal early pregnancy LTPA (hours/week).

^a^ Generalized logistic regression model with normal weight as the reference group.

In general, results for LTPA and sedentary time were similar after additional adjustment for breastfeeding (Supplementary Tables S6 and S7) and occupational activity (physically demanding occupation, Supplementary Tables S8 and S9). Results using 1-hour categories of LTPA as the exposure were also similar to main results using tertiles (Supplementary Table S10).

## Discussion

In this study, we did not observe clear associations of maternal early or late pregnancy LTPA with infant weight status at ∼12 months of age. Our results suggest an association between early pregnancy sedentary time and greater odds of infant underweight at ∼12 months of age. We did not observe differences in associations by infant sex.

Only one previous study, to our knowledge, has examined the association of LTPA during pregnancy with offspring weight in infancy.^23^ Mattran et al. recruited 23 women from prenatal clinics in Michigan and measured infant height, weight, and body fat percentage at 18–24 months old.^23^ At the same follow-up study visit, mothers were asked to recall LTPA during each trimester of pregnancy. In that study, third trimester LTPA was negatively correlated with infant weight (unadjusted Spearman *r* = −0.39) and weight-for-height z-score (unadjusted Spearman *r* = −0.40). LTPA during the first or second trimester was not associated with infant weight at 18–24 months old. We also did not find clear associations of early and late pregnancy LTPA with infant size in the current study.

Some, but not all, studies that have examined associations of maternal LTPA during pregnancy with offspring weight later in childhood (5–8 years of age) have observed associations with lower weight.^13,14,24^ In a retrospective cohort study using nationally representative data on Greek school children, Mourtakos et al. reported that offspring of women who performed any moderate LTPA during pregnancy had 23% lower odds of overweight/obesity at age 8 years than offspring of women who were not physically active during pregnancy.^14^ Clapp also reported that offspring of women who performed regular moderate/vigorous LTPA before pregnancy (runners and aerobic dancers) and remained active throughout pregnancy weighed 0.24 kg less and had 4.6% lower body fat at age 5 years than offspring of women who were active before pregnancy but stopped regular LTPA during pregnancy.^13^ However, in a previous study using the DNBC, Schou Andersen et al. did not observe associations between early or late pregnancy moderate/vigorous LTPA with offspring BMI z-score or overweight status at age 7 years, but this previous study did not include sedentary time.^24^

Previous studies have reported associations of sedentary behavior during pregnancy with proinflammatory biomarkers, such as C-reactive protein (CRP),^25^ and lipid profile, including levels of high-density lipoprotein (HDL).^26^ Higher levels of CRP during pregnancy have been associated with lower birthweight and greater risk for small-for-gestational age.^27^ Higher levels of HDL during pregnancy have been associated with lower birthweight,^28^ decreased risk for macrosomia and large-for-gestational age,^28,29^ and lower infant weight at 6 months.^30^ Taken together, these suggest that maternal sedentary time during pregnancy may affect fetal programming of infant growth by increasing inflammation and altering HDL during pregnancy, which may affect birthweight and subsequent postnatal growth. This is consistent with associations of early pregnancy sedentary time with greater odds of infant underweight observed in our study.

Strengths of this study include its large size and use of prospectively collected data. However, several limitations must be considered. LTPA and sedentary time were self-reported in this study, which may have introduced measurement error. Self-reported information on physical activity in early pregnancy, collected using a questionnaire similar to the one used in our study, had moderate validity (Spearman correlation coefficient = 0.12–0.24) and good reliability (intraclass correlation coefficient = 0.82) compared with accelerometer data recorded among pregnant women.^31^ Results were similar in analyses categorizing LTPA into tertiles and analyses using 1-hour categories of LTPA as the exposure. Measurement of sedentary time was limited to screen time, which may have underestimated total sedentary time. Validity of self-reported time spent watching television among pregnant women has not been assessed; however, validity has been shown to be good compared with accelerometer data (*r* = 0.83) in nonpregnant adults.^32^ Future studies should collect device-based measurements of LTPA and sedentary time to reduce measurement error.

We did not have measurements of infant body composition. We controlled for maternal obesity and smoking in our models, but residual confounding by general healthy lifestyle or maternal and infant illnesses may be present in our results. Women with high sedentary time had similar prevalence of pregnancy complications, such as prepregnancy diabetes, preeclampsia, or gestational diabetes, as women with low sedentary time but potential for residual confounding by unmeasured illness remains. Women with high sedentary time were more likely to be working and have a mostly sitting occupation than women with low sedentary time. Results were similar after adjustment for general occupational activity in sensitivity analyses. We did not include breastfeeding in our main analyses due to the high rate of missingness (33% missing); however, in sensitivity analyses with additional adjustment for categories of any breastfeeding duration, results did not change substantially.

Lifestyle habits present during pregnancy likely continue after pregnancy, reflecting a common lifestyle of the family, which may affect infant postnatal growth. For example, a generally sedentary family could have a more sedentary infant, which may negatively affect muscle development and growth. Prepregnancy lifestyle may also influence associations of LTPA and sitting time during pregnancy with infant outcomes. We did not collect information on physical activity or sitting time in the prepregnancy or postnatal period. Participants in the DNBC were of Danish descent, and most were normal weight and of middle to high socio-occupational status. Results may not be generalizable to more racially and socioeconomically diverse populations.

In conclusion, we did not observe clear associations of maternal LTPA during pregnancy with infant weight at ∼12 months. Our results suggest an association between maternal early pregnancy sedentary time and infant underweight at 12 months. Future studies should confirm results using device-based measurements of physical activity and sedentary behavior, explore associations of changes and patterns of LTPA and sedentary behavior across pregnancy with postnatal growth, as well as investigate associations of maternal sedentary time with later childhood and adult health outcomes.

## Supplementary Material

Supplemental data

Supplemental data

Supplemental data

Supplemental data

Supplemental data

Supplemental data

Supplemental data

Supplemental data

## Abbreviations Used

BMI: body mass index
CIs: confidence intervals
CRP: C-reactive protein
DNBC: Danish National Birth Cohort
HDL: high-density lipoprotein
IQR: interquartile range
LTPA: leisure time physical activity
ORs: odds ratios
SD: standard deviation
