# Supplementary material for: Leisure Time Physical Activity, Sedentary Time in Pregnancy, and Infant Weight at Approximately 12 Months
Source: Womens Health Rep (New Rochelle). 2020 May 12;1(1):123–31. doi: 10.1089/whr.2020.0068 (PMC7325488; doi:10.1089/whr.2020.0068)
Supplement: Supplemental data [file Supp_Table1.pdf]

Supplementary Data

Supplementary Table S1. Maternal and Offspring Characteristics of Analytic Cohort and Excluded Participants, Danish National Birth Cohort

	Excluded participants		Analytic cohort (*N* = 35,212)
**Maternal characteristics**	***N***	**Mean (SD)**	**Mean (SD)**
Age (years)	54,966	29.7 (4.4)	30.2 (4.2)
	N	%	%
Prepregnancy BMI category	47,839		
Underweight (<18.5 kg/m^2)	2,334	5	4
Normal weight (18.5–24.9 kg/m^2)	32,682	68	67
Overweight (25–29.9 kg/m^2)	9,038	19	20
Obese (≥30 kg/m^2)	3,785	8	9
Spouse/partner	49,150	97	99
Socio-occupational status	48,871		
Low	5,107	10	8
Middle	18,045	37	39
High	25,719	53	53
Nulliparous	54,966	46	50
Smoked during pregnancy	29,930	26	24
Gestational diabetes	54,824	1	1
Preeclampsia	54,966	2	2
Infant characteristics	*N*	Mean (SD)	Mean (SD)
Birthweight (g)	54,458	3,576 (577)	3,596 (548)
Gestational age at delivery (weeks)	54,961	39.5 (1.9)	39.6 (1.7)
Weight at interview 4 measurement (kg)	17,087	10.3 (1.3)	10.2 (1.2)
Age at interview 4 measurement (months)	15,824	12.5 (1.7)	12.4 (0.6)
	N	%	%
Male sex	54,869	51	50

BMI, body mass index; SD, standard deviation.
