# Supplementary material for: Leisure Time Physical Activity, Sedentary Time in Pregnancy, and Infant Weight at Approximately 12 Months
Source: Womens Health Rep (New Rochelle). 2020 May 12;1(1):123–31. doi: 10.1089/whr.2020.0068 (PMC7325488; doi:10.1089/whr.2020.0068)
Supplement: Supplemental data [file Supp_Table2.pdf]

Supplementary Table S2. Maternal and Infant Characteristics by Early Pregnancy Leisure Time Physical Activity, Danish National Birth Cohort 1997–2003 (*N* = 35,212)

	No LTPA (0 hour/week)		Low LTPA (<2.5 hours/week)		High LTPA (≥2.5 hours/week)	
**Maternal characteristics**	***N***	**Mean (SD)**	***N***	**Mean (SD)**	***N***	**Mean (SD)**
Age (years)	22,080	30.4 (4.2)	9,062	29.9 (4.0)	4,070	30.0 (4.2)
Gestational weight gain (kg, *N* = 34,959)	21,902	14.9 (6.1)	9,010	14.9 (5.3)	4,047	14.7 (5.4)
Sedentary behavior (hours/day)	22,080	3.1 (2.1)	9,062	3.1 (2.1)	4,070	2.8 (2.0)
	N	%	*N*	%	*N*	%
Prepregnancy BMI category						
Underweight (<18.5 kg/m^2)	988	4	303	3	131	3
Normal weight (18.5–24.9 kg/m^2)	14,454	65	6,294	69	2,951	73
Overweight (25–29.9 kg/m^2)	4,579	21	1,776	20	727	18
Obese (≥30 kg/m^2)	2,059	9	689	8	261	6
Spouse/partner	21,811	99	8,958	99	3,999	98
Socio-occupational status						
Low	2,099	10	558	6	233	6
Middle	9,247	42	3,227	36	1,300	32
High	10,734	49	5,277	58	2,537	62
Employment status						
Employed, working	15,537	70	6,729	74	2,837	70
Employed, on sick leave	1,689	8	365	4	159	4
Employed, on other leave	242	1	77	1	29	1
Student	2,449	11	1,235	14	703	17
Unemployed	2,163	10	656	7	342	8
Nulliparous	9,661	44	5,313	59	2,571	63
Prepregnancy hypertension (*N* = 33,405)	1,085	5	397	5	160	4
Prepregnancy diabetes (*N* = 33,308)	82	0.3	18	0.2	11	0.3
Smoked during pregnancy	6,042	27	1,770	20	785	19
Gestational diabetes	214	1	66	1	29	1
Preeclampsia	496	2	213	2	95	2
Exclusive breastfeeding duration (weeks)						
0–13	5,208	24	1,607	18	703	17
14–21	2,553	12	967	11	432	11
22+	14,319	65	6,488	72	2,935	72
**Infant characteristics**	***N***	**Mean (SD)**	***N***	**Mean (SD)**	***N***	**Mean (SD)**
Birthweight (g)	22,080	3,597 (556)	9,062	3,605 (530)	4,070	3,572 (543)
Gestational age at delivery (weeks)	22,080	39.6 (1.7)	9,062	39.7 (1.6)	4,070	39.6 (1.7)
Weight at interview 4 measurement (kg)	22,080	10.2 (1.2)	9,062	10.2 (1.2)	4,070	10.3 (1.2)
Age at interview 4 measurement (months)	22,080	12.4 (0.6)	9,062	12.4 (0.6)	4,070	12.4 (0.6)
	N	**%**	***N***	**%**	***N***	**%**
Male sex	11,097	50	4,536	50	2,096	52
Weight-for-length category at interview 4 measurement (*n* = 35,143)						
Underweight (<5th percentile)	509	2	217	2	92	2
Normal weight (5th–84th percentile)	15,111	69	6,301	70	2,840	70
Overweight (85th-94th percentile)	3,521	16	1,435	16	608	15
Obese (≥95th percentile)	2,886	13	1,099	12	524	13

LTPA, leisure time physical activity.
