# Supplementary material for: Leisure Time Physical Activity, Sedentary Time in Pregnancy, and Infant Weight at Approximately 12 Months
Source: Womens Health Rep (New Rochelle). 2020 May 12;1(1):123–31. doi: 10.1089/whr.2020.0068 (PMC7325488; doi:10.1089/whr.2020.0068)
Supplement: Supplemental data [file Supp_Table3.pdf]

Supplementary Table S3. Maternal and Infant Characteristics by Early Pregnancy Sedentary Time, Danish National Birth Cohort 1997–2003 (*N* = 35,212)

	Low sedentary time (0 to <2 hours/day)		Moderate sedentary time (2 to <5 hours/day)		High sedentary time (≥5 hours/day)	
**Maternal characteristics**	***N***	**Mean (SD)**	***N***	**Mean (SD)**	***N***	**Mean (SD)**
Age (years)	11,085	30.2 (4.3)	16,569	30.0 (4.1)	7,558	30.5 (3.8)
Early pregnancy moderate/vigorous leisure time physical activity (hours/week)	11,085	0.9 (1.7)	16,569	0.8 (1.7)	7,558	0.7 (1.4)
Late pregnancy moderate/vigorous leisure time physical activity (hours/week)	11,056	0.5 (1.2)	16,531	0.4 (1.0)	7,543	0.4 (1.0)
Gestational weight gain (kg, *N* = 34,959)	10,993	14.8 (5.6)	16,458	15.0 (6.0)	7,508	14.8 (5.7)
	N	%	***N***	**%**	***N***	**%**
Any early pregnancy moderate/vigorous leisure time physical activity	4,318	39	6,058	37	2,756	36
Any late pregnancy moderate/vigorous leisure time physical activity	3,001	27	3,902	24	1,827	24
Prepregnancy BMI category						
Underweight (<18.5 kg/m^2)	463	4	633	4	326	4
Normal weight (18.5–24.9 kg/m^2)	7,809	70	10,848	65	5,042	67
Overweight (25–29.9 kg/m^2)	2,029	18	3,499	21	1,554	21
Obese (≥30 kg/m^2)	1,142	7	1,589	10	636	8
Spouse/partner	10,935	99	16,358	99	7,475	99
Socio-occupational status						
Low	960	9	1,638	10	292	4
Middle	3,397	31	6,645	40	3,732	49
High	6,728	61	8,286	50	3,534	47
Employed, working	6,977	63	11,286	68	6,840	91
Employed, on sick leave	567	5	1,220	7	426	6
Employed, on other leave	155	1	178	1	15	0.2
Student	2,288	21	2,020	12	79	1
Unemployed	1,098	10	1,865	11	198	3
Physically demanding occupation (among employed women, *N* = 27,142)	2,327	31	3,087	25	314	4
Mostly sitting occupation (among employed women, *N* = 26,987)	384	5	2,200	18	4,219	58
Nulliparous	5,041	45	8,263	50	4,241	56
Prepregnancy hypertension (*N* = 33,405)	442	4	812	5	361	5
Prepregnancy diabetes (*N* = 33,308)	38	0.3	57	0.4	16	0.2
Smoked during pregnancy	2,479	22	4,373	26	1,745	23
Gestational diabetes	82	1	160	1	67	1
Preeclampsia	227	2	408	2	169	2
Exclusive breastfeeding duration (weeks, *N* = 35,424)						
0–13	2,123	19	3,807	23	1,588	21
14–21	1,074	10	1,930	12	948	13
22+	7,888	71	10,832	65	5,022	66
Infant characteristics	***N***	**Mean (SD)**	***N***	**Mean (SD)**	***N***	**Mean (SD)**
Birthweight (g)	17,633	3,613 (539)	15,811	3,587 (552)	1,950	3,522 (591)
Gestational age at delivery (weeks)	17,633	39.6 (1.6)	15,811	39.6 (1.7)	1,950	39.5 (1.8)
Weight at interview 4 measurement (kg)	17,633	10.3 (1.2)	15,811	10.2 (1.2)	1,950	10.2 (1.3)
Age at interview 4 measurement (months)	17,633	12.4 (0.6)	15,811	12.4 (0.6)	1,950	12.4 (0.6)
	N	**%**	***N***	**%**	***N***	**%**
Male sex	5,647	51	8,284	50	3,798	50
Weight-for-length category at interview 4 measurement (*n* = 35,143)						
Underweight (<5th percentile)	233	2	402	2	183	2
Normal weight (5th–84th percentile)	7,642	69	11,380	69	5,230	69
Overweight (85th–94th percentile)	1,758	16	2,603	16	1,203	16
Obese (≥95th percentile)	1,431	13	2,148	13	930	12
