# Supplementary material for: Leisure Time Physical Activity, Sedentary Time in Pregnancy, and Infant Weight at Approximately 12 Months
Source: Womens Health Rep (New Rochelle). 2020 May 12;1(1):123–31. doi: 10.1089/whr.2020.0068 (PMC7325488; doi:10.1089/whr.2020.0068)
Supplement: Supplemental data [file Supp_Tables4-5.pdf]

**Supplementary Table S4. Associations of Late Pregnancy Leisure Time Physical Activity with Infant Weight at ~12 Months**

| | Weight (kg) adjusted for length (cm) | | Underweight (<5th percentile) | | Normal weight (5–84th percentile) | | Overweight (85–94th percentile) | | Obese (≥95th percentile) | |
|---|---|---|---|---|---|---|---|---|---|---|
| | *N* | Mean difference (95% CI)[a] | N | OR (95% CI)[a,b] | *N* | OR (95% CI)[a,b] | *N* | OR (95% CI)[a,b] | *N* | OR (95% CI)[a,b] |
| Continuous (hours/week) | 35,130 | 0.00 (−0.01 to 0.01) | 807 | 0.98 (0.91 to 1.05) | 24,196 | Ref. | 5,557 | 1.00 (0.98 to 1.01) | 4,501 | 0.99 (0.98 to 1.01) |
| No physical activity | 26,400 | Ref. | 606 | Ref. | 18,123 | Ref. | 4,234 | Ref. | 3,381 | Ref. |
| Tertile 1 (0.03–0.93) | 2,537 | −0.01 (−0.05 to 0.03) | 64 | 1.08 (0.83 to 1.40) | 1,750 | Ref. | 395 | 0.97 (0.87 to 1.09) | 315 | 0.99 (0.87 to 1.12) |
| Tertile 2 (1.00–1.58) | 3,291 | 0.00 (−0.04 to 0.04) | 71 | 0.92 (0.72 to 1.19) | 2,307 | Ref. | 481 | 0.90 (0.81 to 1.00) | 429 | 1.02 (0.91 to 1.14) |
| Tertile 3 (1.63–29) | 2,910 | 0.00 (−0.03 to 0.04) | 66 | 0.99 (0.76 to 1.28) | 2,016 | Ref. | 447 | 0.96 (0.86 to 1.07) | 376 | 1.04 (0.92 to 1.17) |
| *p* for trend | | 0.91 | | 0.73 | | | | 0.11 | | 0.56 |

*p* for interaction with offspring sex: continuous LTPA: weight $p=0.75$; weight categories $p=0.67$; LTPA tertiles: weight $p=0.87$; weight categories $p=0.35$.

[a]Model is adjusted for maternal age (years), prepregnancy BMI category (underweight/normal weight/overweight/obese), nulliparity (yes/no), smoking during pregnancy (yes/no), spouse/partner (yes/no), socio-occupational status (high/middle/low), employment (working/on sick leave/on other leave/student/unemployed), total sedentary time (hours/day), infant age at interview 4 measurement (months), infant length at interview 4 measurement (cm), and infant sex.

[b]Generalized logistic regression model with normal weight as the reference group.

CI, confidence interval; OR, odds ratio.

**Supplementary Table S5. Associations of Early Pregnancy Leisure Time Physical Activity with Infant Weight at ~12 Months Additionally Adjusted for Late Pregnancy Physical Activity**

| | Weight (kg) adjusted for length (cm) | | Underweight (<5th percentile) | | Normal weight (5–84th percentile) | | Overweight (85–94th percentile) | | Obese (≥95th percentile) | |
|---|---|---|---|---|---|---|---|---|---|---|
| Model[a] | *N* | Mean difference (95% CI) | *N* | OR (95% CI)[b] | *N* | OR (95% CI)[b] | *N* | OR (95% CI)[b] | *N* | OR (95% CI)[b] |
| Continuous (hours/week) | 35,130 | 0.00 (−0.01 to 0.00) | 807 | 1.00 (0.95 to 1.04) | 24,196 | Ref. | 5,557 | 0.99 (0.97 to 1.01) | 4,501 | 0.99 (0.97 to 1.01) |
| No physical activity | 22,028 | Ref. | 503 | Ref. | 15,074 | Ref. | 3,517 | Ref. | 2,881 | Ref. |
| Tertile 1 (0.01–1.00) | 4,993 | 0.00 (−0.03 to 0.03) | 122 | 1.07 (0.87 to 1.31) | 3,429 | Ref. | 821 | 1.04 (0.96 to 1.14) | 618 | 0.95 (0.86 to 1.05) |
| Tertile 2 (1.01–2.17) | 3,743 | −0.04 (−0.08 to 0.00) | 82 | 0.93 (0.73 to 1.18) | 2,653 | Ref. | 561 | 0.92 (0.83 to 1.02) | 440 | 0.87 (0.78 to 0.97) |
| Tertile 3 (2.18–30) | 4,366 | 0.01 (−0.03 to 0.04) | 100 | 0.99 (0.79 to 1.08) | 3,040 | Ref. | 658 | 0.95 (0.86 to 1.04) | 562 | 0.98 (0.88 to 1.08) |
| *p* for trend | | 0.59 | | 0.80 | | | | 0.15 | | 0.15 |

*p* for interaction with offspring sex: continuous LTPA: weight $p=0.70$; weight categories $p=0.55$; LTPA tertiles: weight $p=0.91$; weight categories $p=0.97$.

[a]Model is adjusted for maternal age (years), prepregnancy BMI category (underweight/normal weight/overweight/obese), nulliparity (yes/no), smoking during pregnancy (yes/no), spouse/partner (yes/no), socio-occupational status (high/middle/low), employment (working/on sick leave/on other leave/student/unemployed), total sedentary time (hours/day), late pregnancy leisure time physical activity (hours/week), infant age at interview 4 measurement (months), infant length at interview 4 measurement (cm), and infant sex.

[b]Model is a generalized logistic regression model with normal weight as the reference group.
