# Supplementary material for: Leisure Time Physical Activity, Sedentary Time in Pregnancy, and Infant Weight at Approximately 12 Months
Source: Womens Health Rep (New Rochelle). 2020 May 12;1(1):123–31. doi: 10.1089/whr.2020.0068 (PMC7325488; doi:10.1089/whr.2020.0068)
Supplement: Supplemental data [file Supp_Table6.pdf]

Supplementary Table S6. Associations of Early Pregnancy Leisure Time Physical Activity (Hours/Week) with Infant Weight at ~12 Months Additionally Adjusted for Breastfeeding

	Weight (kg) adjusted for length (cm)		Underweight (<5th percentile)		Normal weight (5–85th percentile)		Overweight (85–95th percentile)		Obese (≥95th percentile)	
Model[a]	*N*	Mean difference (95% CI)	*N*	OR (95% CI)[b]	*N*	OR (95% CI)[b]	*N*	OR (95% CI)[b]	*N*	OR (95% CI)[b]
Continuous (hours/week)	35,212	0.00 (−0.01 to 0.00)	818	0.99 (0.95 to 1.04)	24,252	Ref.	5,564	0.98 (0.97 to 1.00)	4,509	0.99 (0.97 to 1.01)
No physical activity	22,080	Ref.	509	Ref.	15,111	Ref.	3,521	Ref.	2,886	Ref.
Tertile 1 (0.01–1.00)	5,003	0.00 (−0.03 to 0.03)	125	1.08 (0.89 to 1.33)	3,434	Ref.	821	1.04 (0.95 to 1.13)	620	0.97 (0.88 to 1.06)
Tertile 2 (1.05–2.23)	3,752	−0.04 (−0.07 to 0.00)	83	0.93 (0.73 to 1.18)	2,659	Ref.	562	0.92 (0.83 to 1.01)	441	0.89 (0.79 to 0.99)
Tertile 3 (2.25–30)	4,377	0.01 (−0.02 to 0.05)	101	0.98 (0.79 to 1.23)	3,048	Ref.	660	0.94 (0.86 to 1.03)	562	0.99 (0.90 to 1.10)
p for trend		0.79		0.75				0.09		0.29

p for interaction with offspring sex: continuous LTPA: weight $p = 0.77$; weight categories $p = 0.58$; LTPA tertiles: weight $p = 0.91$; weight categories $p = 0.98$.

[a]Model is adjusted for maternal age (years), prepregnancy BMI category (underweight/normal weight/overweight/obese), nulliparity (yes/no), smoking during pregnancy (yes/no), spouse/partner (yes/no), socio-occupational status (high/middle/low), employment (working/on sick leave/on other leave/student/unemployed), total sedentary time (hours/day), total breastfeeding duration (0–13 weeks/14–21 weeks/22+ weeks), infant age at interview 4 measurement (months), infant length at interview 4 measurement (cm), and infant sex.

[b]Model is a generalized logistic regression model with normal weight as the reference group.
