# Supplementary material for: Leisure Time Physical Activity, Sedentary Time in Pregnancy, and Infant Weight at Approximately 12 Months
Source: Womens Health Rep (New Rochelle). 2020 May 12;1(1):123–31. doi: 10.1089/whr.2020.0068 (PMC7325488; doi:10.1089/whr.2020.0068)
Supplement: Supplemental data [file Supp_Table7.pdf]

Supplementary Table S7. Associations of Early Pregnancy Sedentary Time with Infant Weight at ~12 Months Additionally Adjusted for Breastfeeding

	Weight (kg) adjusted for length (cm)		Underweight (<5th percentile)		Normal weight (5–84th percentile)		Overweight (85–94th percentile)		Obese (≥95th percentile)	
Model[a]	*N*	Mean difference (95% CI)	*N*	OR (95% CI)[b]	*N*	OR (95% CI)[b]	*N*	OR (95% CI)[b]	*N*	OR (95% CI)[b]
Continuous (hours/ day)	35,212	0.00 (−0.01 to 0.00)	818	1.01 (0.98 to 1.05)	24,252	Ref.	5,564	1.00 (0.98 to 1.01)	4,509	0.99 (0.98 to 1.01)
Quartile 1 (0–1.4)	7,978	Ref.	163	Ref.	5,520	Ref.	1,278	Ref.	1,001	Ref.
Quartile 2 (1.5–2.3)	9,636	−0.01 (−0.04 to 0.02)	245	1.27 (1.03 to 1.55)	6,614	Ref.	1,509	0.96 (0.89 to 1.05)	1,249	1.01 (0.92 to 1.10)
Quartile 3 (2.4–4.5)	8,719	−0.01 (−0.04 to 0.02)	200	1.15 (0.93 to 1.42)	5,998	Ref.	1,342	0.94 (0.86 to 1.02)	1,160	1.04 (0.95 to 1.14)
Quartile 4 (4.6–10.7)	8,879	−0.02 (−0.05 to 0.02)	210	1.19 (0.96 to 1.48)	6,120	Ref.	1,435	0.98 (0.90 to 1.07)	1,099	0.99 (0.89 to 1.09)
p for trend		0.38		0.28				0.60		0.92

p for interaction with infant sex: continuous sedentary time: weight $p = 0.62$; weight categories $p = 0.92$; sedentary time quartiles: weight $p = 0.03$; weight categories $p = 0.07$.

[a]Model is adjusted for maternal age (years), prepregnancy BMI category (underweight/normal weight/overweight/obese), nulliparity (yes/no), smoking during pregnancy (yes/no), spouse/partner (yes/no), socio-occupational status (high/middle/low), infant sex, infant age at interview 4 measurement (months), and maternal early pregnancy leisure time physical activity (hours/week).

[b]Generalized logistic regression model with normal weight as the reference group.
