# Supplementary material for: Leisure Time Physical Activity, Sedentary Time in Pregnancy, and Infant Weight at Approximately 12 Months
Source: Womens Health Rep (New Rochelle). 2020 May 12;1(1):123–31. doi: 10.1089/whr.2020.0068 (PMC7325488; doi:10.1089/whr.2020.0068)
Supplement: Supplemental data [file Supp_Tables8-9.pdf]

Supplementary Table S8. Associations of Early Pregnancy Leisure Time Physical Activity with Infant Weight at ~12 Months Additionally Adjusted for Occupational Activity

	Weight (kg) adjusted for length (cm)		Underweight (<5th percentile)		Normal weight (5–84th percentile)		Overweight (85–94th percentile)		Obese (≥95th percentile)	
Model[a]	*N*	Mean difference (95% CI)	*N*	OR (95% CI)[b]	*N*	OR (95% CI)[b]	*N*	OR (95% CI)[b]	*N*	OR (95% CI)[b]
Continuous (hours/week)	64,690	0.00 (−0.01 to 0.00)	801	0.99 (0.95 to 1.04)	23,889	Ref.	5,491	0.99 (0.97 to 1.00)	4,440	0.99 (0.97 to 1.01)
No physical activity	21,696	Ref.	495	Ref.	14,853	Ref.	3,463	Ref.	2,832	Ref.
Tertile 1 (0.01–1.00)	4,955	0.00 (−0.03 to 0.03)	125	1.11 (0.90 to 1.35)	3,398	Ref.	816	1.04 (0.95 to 1.13)	613	0.96 (0.87 to 1.06)
Tertile 2 (1.05–2.23)	3,711	−0.04 (−0.07 to 0.00)	82	0.94 (0.74 to 1.19)	2,626	Ref.	558	0.92 (0.83 to 1.01)	438	0.89 (0.79 to 0.99)
Tertile 3 (2.25–30)	4,328	0.01 (−0.02 to 0.04)	99	0.99 (0.79 to 1.23)	3,012	Ref.	654	0.94 (0.86 to 1.04)	557	0.99 (0.90 to 1.10)
p for trend		0.73		0.80				0.10		0.27

[a]Model is adjusted for maternal age (years), prepregnancy BMI category (underweight/normal weight/overweight/obese), nulliparity (yes/no), smoking during pregnancy (yes/no), spouse/partner (yes/no), socio-occupational status (high/middle/low), employment (working/on sick leave/on other leave/student/unemployed), physically demanding job (yes/no), total sedentary time (hours/day), infant age at interview 4 measurement (months), infant length at interview 4 measurement (cm), and infant sex.

[b]Generalized logistic regression model with normal weight as the reference group.

Supplementary Table S9. Associations of Early Pregnancy Sedentary Time with Infant Weight at ~12 Months Additionally Adjusted for Occupational Activity

	Weight (kg) adjusted for length (cm)		Underweight (<5th percentile)		Normal weight (5–84th percentile)		Overweight (85–94th percentile)		Obese (≥95th percentile)	
Model[a]	*N*	Mean difference (95% CI)	*N*	OR (95% CI)[b]	*N*	OR (95% CI)[b]	*N*	OR (95% CI)[b]	*N*	OR (95% CI)[b]
Continuous (hours/day)	64,690	0.00 (−0.01 to 0.00)	801	1.02 (0.98 to 1.06)	23,889	Ref.	5,491	0.99 (0.98 to 1.01)	4,440	0.99 (0.97 to 1.01)
Quartile 1 (0–1.4)	7,827	Ref.	160	Ref.	5,408	Ref.	1,262	Ref.	981	Ref.
Quartile 2 (1.5–2.3)	9,439	−0.01 (−0.04 to 0.02)	237	1.25 (1.02 to 1.53)	6,482	Ref.	1,478	0.96 (0.88 to 1.04)	1,223	1.01 (0.92 to 1.11)
Quartile 3 (2.4–4.5)	8,578	−0.01 (−0.04 to 0.02)	195	1.14 (0.92 to 1.42)	5,899	Ref.	1,322	0.93 (0.85 to 1.01)	1,143	1.05 (0.95 to 1.15)
Quartile 4 (4.6–10.7)	8,846	−0.02 (−0.05 to 0.01)	209	1.21 (0.96 to 1.52)	6,100	Ref.	1,429	0.95 (0.87 to 1.05)	1,093	0.99 (0.89 to 1.09)
p for trend		0.27		0.22				0.25		0.98

[a]Model is adjusted for maternal age (years), prepregnancy BMI category (underweight/normal weight/overweight/obese), nulliparity (yes/no), smoking during pregnancy (yes/no), spouse/partner (yes/no), socio-occupational status (high/middle/low), employment (working/on sick leave/on other leave/student/unemployed), physically demanding job (yes/no), total sedentary time (hours/day), infant age at interview 4 measurement (months), infant length at interview 4 measurement (cm), and infant sex.

[b]Generalized logistic regression model with normal weight as the reference group.
