# Supplementary material for: Leisure Time Physical Activity, Sedentary Time in Pregnancy, and Infant Weight at Approximately 12 Months
Source: Womens Health Rep (New Rochelle). 2020 May 12;1(1):123–31. doi: 10.1089/whr.2020.0068 (PMC7325488; doi:10.1089/whr.2020.0068)
Supplement: Supplemental data [file Supp_Table10.pdf]

Supplementary Table S10. Associations of Early Pregnancy Leisure Time Physical Activity (Hours/Week) with Infant Weight at ~12 Months

	Weight (kg) adjusted for length (cm)		Underweight (<5th percentile)		Normal weight (5–84th percentile)		Overweight (85–94th percentile)		Obese (≥95th percentile)	
Model[a]	*N*	Mean difference (95% CI)	*N*	OR (95% CI)[b]	*N*	OR (95% CI)[b]	*N*	OR (95% CI)[b]	*N*	OR (95% CI)[b]
No physical activity	22,080	Ref.	509	Ref.	15,111	Ref.	3,521	Ref.	2,886	Ref.
0.01–1.00 hours/week	5,003	0.00 (−0.03 to 0.03)	125	1.08 (0.89 to 1.32)	3,434	Ref.	821	1.03 (0.95 to 1.12)	620	0.96 (0.87 to 1.06)
1.01–2.00 hours/week	3,636	−0.04 (−0.07 to 0.00)	79	0.90 (0.71 to 1.15)	2,587	Ref.	537	0.90 (0.81 to 0.99)	426	0.87 (0.78 to 0.98)
2.01–3.00 hours/week	1,925	0.02 (−0.03 to 0.06)	46	1.04 (0.76 to 1.41)	1,320	Ref.	315	1.03 (0.91 to 1.17)	242	0.97 (0.84 to 1.12)
3.01–4.00 hours/week	1,078	0.05 (−0.01 to 0.11)	24	0.96 (0.63 to 1.46)	740	Ref.	158	0.93 (0.78 to 1.11)	155	1.13 (0.95 to 1.36)
4.01–5.00 hours/week	619	−0.04 (−0.12 to 0.04)	17	1.15 (0.70 to 1.89)	437	Ref.	90	0.90 (0.71 to 1.13)	74	0.92 (0.71 to 1.18)
5.01–6.00 hours/week	341	−0.07 (−0.18 to 0.04)	8	0.93 (0.46 to 1.89)	251	Ref.	43	0.75 (0.54 to 1.03)	39	0.83 (0.59 to 1.17)
>6.00 hours/week	530	−0.03 (−0.11 to 0.06)	10	0.80 (0.42 to 1.51)	372	Ref.	79	0.92 (0.72 to 1.17)	67	0.95 (0.73 to 1.23)
p for trend		0.46		0.68				0.05		0.30

[a]Model is adjusted for maternal age (years), prepregnancy BMI category (underweight/normal weight/overweight/obese), nulliparity (yes/no), smoking during pregnancy (yes/no), spouse/partner (yes/no), socio-occupational status (high/middle/low), employment (working/on sick leave/on other leave/student/unemployed), total sedentary time (hours/day), infant age at interview 4 measurement (months), infant length at interview 4 measurement (cm), and infant sex.

[b]Generalized logistic regression model with normal weight as the reference group.
